# DTI Measurements in Multiple Sclerosis: Evaluation of Brain Damage and Clinical Implications

**DOI:** 10.1155/2013/671730

**Published:** 2013-03-31

**Authors:** Emilia Sbardella, Francesca Tona, Nikolaos Petsas, Patrizia Pantano

**Affiliations:** Department of Neurology and Psychiatry, Sapienza University, Viale dell'Università 30, 00185 Rome, Italy

## Abstract

Diffusion tensor imaging (DTI) is an effective means of quantifying parameters of demyelination and axonal loss. The application of DTI in Multiple Sclerosis (MS) has yielded noteworthy results. DTI abnormalities, which are already detectable in patients with clinically isolated syndrome (CIS), become more pronounced as disease duration and neurological impairment increase. The assessment of the microstructural alterations of white and grey matter in MS may shed light on mechanisms responsible for irreversible disability accumulation. In this paper, we examine the DTI analysis methods, the results obtained in the various tissues of the central nervous system, and correlations with clinical features and other MRI parameters. The adoption of DTI metrics to assess the outcome of prognostic measures may represent an extremely important step forward in the MS research field.

## 1. Introduction

In multiple sclerosis (MS) research, nonconventional magnetic resonance imaging (MRI) techniques have demonstrated a high degree of specificity and sensitivity in detecting pathological tissue damage [1]. These techniques include diffusion-weighted imaging, which plays an important role in highlighting brain microstructural damage not visible when conventional sequences are used. Diffusion imaging principles are based on the measurement of motion of water molecules within tissues [2]. Free water usually moves equally in all directions in an isotropic fashion; when, however, water is restricted inside or by tissues, preferential directions are taken and movement consequently becomes anisotropic. Therefore, water mobility in the brain is markedly reduced in compact tissue, such as white matter (WM), is reduced to a lesser extent in the grey matter (GM), and is almost free in the cerebrospinal fluid (CSF). Pathological processes that alter the normal brain structure may affect water motion, with effects on the resulting diffusion indexes.

**Figure 1:**
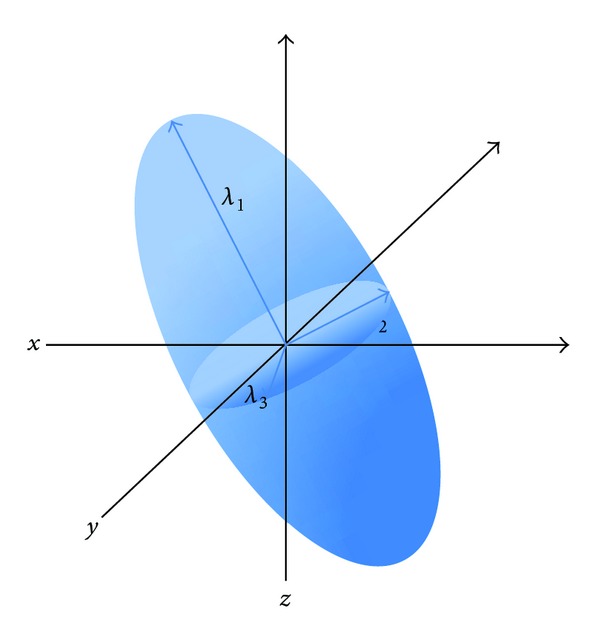
Elliptical representation of a diffusion tensor with the 3 main axes: the longest axis stands for the primary eigenvector (*λ*_1_), reflecting the diffusion parallel to the fibers; the two shorter axes represent the second (*λ*_2_) and third (*λ*_3_) eigenvectors, whose average provides a measure of diffusivity perpendicular to the fibers.

Diffusion images can be acquired from a minimum of three gradient directions, which yield two different kinds of sequences: diffusion-weighted imaging (DWI) and diffusion tensor imaging (DTI), respectively. The diffusion tensor is a matrix acquired from at least 6 gradient directions that characterizes three-dimensional water movement. It can be represented as an ellipsoid whose components are 3 main axes [3] (Figure 1): the longest axis stands for the primary eigenvector (*λ*_1_) and reflects diffusion parallel to the fibers, or axial diffusivity (AD); the two shorter axes represent the second (*λ*_2_) and third (*λ*_3_) eigenvectors and are averaged to provide a measure of diffusivity perpendicular to the fibers, or radial diffusivity (RD) [4]. The aforementioned metrics have proved to be able to discriminate between axonal damage and demyelinated damage: the former is better expressed by AD, a measure of axonal integrity, and the latter by RD, a measure of myelin integrity [5–8] (Table 1). Indeed, the diffusion study of shiverer mice, which have incomplete myelin formation in the CNS not accompanied by inflammation or axonal injury processes, helped to better define the meaning of AD and RD [5]. The authors of that study demonstrated that RD was significantly higher in shiverer mice than in controls, whilst AD was unchanged, thus pointing to RD and AD as markers of myelin and axonal integrity, respectively.

In WM tracts of MS patients, RD is typically increased owing to the loss of myelin, whereas AD has been reported to be either increased or decreased in comparison with healthy subjects (HS) [6]. A decrease in AD may be the consequence of axonal loss, whereas an increase has been interpreted as an attempt made by a compensative mechanism to maintain functionality in the presence of WM damage [6]. 

The DTI metrics used most, derived from a mathematical combination of the three eigenvectors [7], are mean diffusivity (MD), which measures overall water motion without any directionality, and fractional anisotropy (FA), which reflects the prevalence of diffusivity along one direction [8]. MD is a quantitative metric of water diffusion; the higher the MD value, the higher the diffusivity. FA is a scalar value ranging from 0 to 1 that is highest in compact WM tracts, decreases in the GM, and approaches zero in the CSF [8] (Table 1). In early studies, anisotropy was correlated to axon density and myelin content, while diffusivity was correlated above all to the amount of myelin [9]. However, both MD and FA have more recently been shown to be affected mainly by myelin content [10] and, to a lesser extent, by axonal density [11]. Briefly, MD has so far been interpreted as an index that is primarily influenced by free space, which means that processes such as vasogenic edema, axonal, and myelin loss increase its value [12]. On the other hand, FA is believed to be more sensitive to the detection of the integrity of WM, as significant FA differences have been observed between myelinated and nonmyelinated nerves [13–16]. Nonetheless, FA is not very specific and does not distinguish between diseases characterized by a range of pathological processes, such as edema, inflammation, demyelination, and leukoaraiosis [17]. 

The idea of diffusion MRI was introduced in 1986 [2] and was subsequently applied to the study of a number of neurological diseases, including MS [18]. Since it was first applied, methods of diffusion analyses have been steadily improved. Although region of interest (ROI) analysis was adopted in early MS studies [12, 14, 18–21], this method proved to have some drawbacks; that is, it is time-consuming, operator dependent, and subject to partial volume artifacts; furthermore, it does not provide a global assessment of tissue damage [22]. Accordingly, histogram analyses were carried out to evaluate the diffusion metrics in the whole brain [22–24]. Subsequently, a color was attributed to diffusion direction along each of the three planes of space, thus providing information on the direction of WM tracts [25].

Finally, in order to respond to a growing interest in the identification of regional diffusion changes, a whole brain voxel-based morphometry (VBM) approach [26, 27], usually applied to T1-weighted images to study atrophy, was used for diffusion images. However, owing to the limitations of this technique in terms of alignment inaccuracies and smoothing extent, VBM was partially superseded by tract-based spatial statistics (TBSS), a fully automated, whole brain diffusion analysis method [28]. 

**Figure 2:**
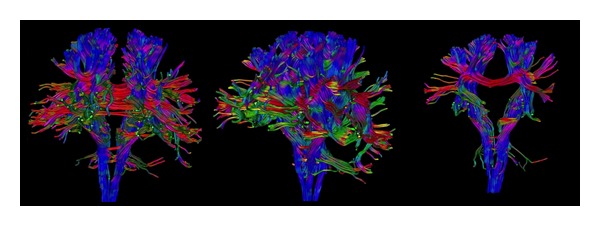
Three-dimensional tractography that reconstructs brain white matter bundles. The different colors represent different directions of the fibers.

Another way of studying diffusion images is based on the reconstruction of large fiber bundles using three-dimensional tractography [29–32] (Figure 2). This method delimits major tracts of WM *in vivo*: after the selection of one, or more than one, seed ROI, nervous pathways are reconstructed by tracking along the principal direction of the fibers passing through the ROI [33]. This technique can be used to analyze the displacement of fibers as well as to detect Wallerian degeneration [34]. It does, however, have some limitations, that is, difficulties to find the principal direction when there are many crossing fibers, low signal-to-noise ratio, poor spatial resolution, nondetectable small fibers, and difficulties in case of distorted brain, all of which require an a priori knowledge of anatomical structures [35]. To partially overcome these problems, the use of more sophisticated mathematical models [36], constraints on the fiber tracking [37–39], and use of WM probability maps from HS [40] have been proposed.

## 2. Diffusion Studies in Different MS Tissues 

Since diffusion MRI was first introduced, many works have applied DWI, and particularly DTI, to study patients affected by MS. Various methodological approaches of DTI analyses have been used to characterize different types of tissue damage. In this section, we describe the DTI results obtained in different types of brain and spinal cord tissues. 

### 2.1. Normal Appearing White Matter (NAWM)

In MS patients, widespread DTI abnormalities, consisting of increased MD and decreased FA, have been detected in NAWM [1, 12, 19, 23, 41–44]. A FA change gradient has also been demonstrated, with lower values being observed close to the plaques and higher values far from the plaques [44]. Histological studies, which have detected demyelination and axon transection even in WM outside the plaques [10, 45], suggest that DTI changes in NAWM may be ascribed to Wallerian degeneration processes [46].

Abnormalities in NAWM have been detected in almost all MS phenotypes, though the degree of damage varies according to the severity of the pathology. Indeed, although altered NAWM is visible from the onset of clinical symptoms, even at an early age, DTI-detectable damage becomes increasingly evident as the disease worsens.

Disability is less pronounced and pathological tissue damage is less severe in children with MS and in patients with clinically isolated syndrome (CIS) than in other phenotypes. In pediatric patients with CIS, at the very onset of disease, the NAWM diffusion metrics appear to be normal if compared with HS [47]. By contrast, increased RD and MD [27, 48, 49] and decreased FA and AD [50] values are found in most WM tracts in pediatric patients with definite MS and in adult patients with CIS compared with HS [47, 50–54]. Although agreement amongst researchers is not unanimous [27, 40, 55], these changes have been interpreted as a sign of early fiber loss in WM. Inconsistencies exist with regard to FA results, as some studies did not detect any changes. This may be explained by the fact that axonal transaction may, despite being present from disease onset [56], affect patients in different ways in the early stages and escape detection by DTI in the very early phases. By contrast, as the pathology progresses, the structural damage becomes more pronounced. Indeed, changes in NAWM are less evident in CIS than in patients with definite MS [27, 57]. Likewise, in patients with benign MS, FA and AD have been shown to be higher than those in relapsing remitting (RR) MS patients [27], although alterations in other DTI indexes, that is, MD and RD [58], have not been detected. This discrepancy points to the existence of neuroprotective mechanisms that may prevent axonal injury [27, 59] and clinical worsening, despite the inflammatory process. Moreover, DTI metrics have revealed differences between benign and RRMS patients in the topographical distribution of WM damage, which might be associated with the favorable clinical status in the former group [59].

Most [27], though not all [24, 60], studies, have detected differences in NAWM diffusion metrics between RRMS patients and HS. The lack of changes in RRMS observed in some studies is likely to be attributable either to the fact that subtle damage is not easily detected in the early phase of the disease or to the poor sensitivity of the ROI methodology adopted in those studies. 

In comparison with CIS, RR, and benign MS, secondary progressive (SP) MS patients exhibit more pronounced WM diffusion abnormalities [27, 61, 62]. The greater increase in diffusivity in SPMS than in other phenotypes may represent a more advanced phase of the disease, presumably characterized by a combination of axonal loss and tissue destruction processes with inflammatory events [62]. The high degree of axonal degeneration in such patients is confirmed by the widespread decrease in FA, not only within lesions but also in NAWM [27]. 

Although widespread diffusivity changes, consisting of increased MD, RD, and AD and decreased FA, have been demonstrated in primary progressive (PP) MS if compared with HS [27, 63], NAWM is affected to a lesser degree in PPMS patients than in those with SPMS [27, 64], probably owing to the more pronounced inflammation present in the SP than in the PP phenotype.

### 2.2. Normal Appearing Grey Matter (NAGM)

Besides involving the WM, MS also affects the GM, with microscopic damage being detectable even in the absence of macroscopic lesions [65, 66]. Anatomical changes are usually visible in the deep [67–69] and cortical GM [69, 70] in most MS phenotypes [64, 71–73]. However, some studies using ROI analysis did not provide conclusive evidence regarding the presence of NAGM abnormalities [60, 72], which suggests that different analysis methods might yield contrasting results.

The fact that no DTI changes have been detected in the NAGM of early onset patients [51] indicates that the GM might be preserved at an early age. However, the pathological involvement of NAGM was demonstrated when adult patients with CIS were studied [54, 57], with increased MD and decreased FA being detected in such patients, though to a less severe degree than in RRMS patients [54, 57]. 

Adults with benign MS have also exhibited changes in DTI [19, 21, 74], consisting in particular of an increase in MD [75], which thus suggests that demyelination prevails over axonal damage when the disease is clinically less severe. 

Most recent works have detected abnormalities, consisting of both FA and MD [42, 76] changes, in the cortical or subcortical NAGM of RRMS patients compared with controls [27]. While MD usually increases, FA has been found to either increase [42, 77] or decrease [76]. This discrepancy may be explained by the phase of GM inflammation at the time of the analysis, with the prevailing activation in GM of microglia or inflammatory processes leading, respectively, to an increased or decreased FA [78]. Other authors have instead reported no diffusion abnormalities in patients with early MS when compared with controls [1, 60, 77]. The reasons for this discrepancy may be the use of ROIs, the improvement in processing steps, and the heterogeneity of the sample size.

SPMS patients display more pronounced GM diffusion abnormalities [27, 54, 64, 71], at both the deep and cortical levels [69], than other MS phenotypes, probably owing to the concomitant presence of a high degree of inflammation and degeneration in this group of patients. 

### 2.3. Spinal Cord (SC)

It has been demonstrated that NAWM and T2 lesions within the SC yield increased AD and RD and decreased FA if compared with normal tissues. These abnormalities have been correlated with the degree of demyelination [79], while FA has also been closely correlated with axonal density [9]. Given the high sensitivity displayed by RD to discriminate myelin content, a recent study proposed increased RD as a marker of increasing severity of demyelination [80]. 

When cervical cord was assessed in benign MS patients, few abnormalities, consisting of increased MD, were detected outside focal macroscopic lesions [81]. Similarly, patients with early onset MS exhibited a slight increase in the MD of the NAWM, which points to a mild degree of damage and explains the more favorable clinical course in early onset than in adult onset patients [82]. 

Conversely, reduced FA and increased MD were observed in NAWM and in surrounding demyelinating plaques [81, 83–86] in RRMS patients as well as in the NAWM of patients without any lesions within the whole SC [87]. This finding points to widespread pathological involvement of the spine, regardless of the presence of T2 lesions. Subjects with a progressive phenotype, that is, both PP and SP patients, also displayed increased MD and decreased FA in the NAWM of cervical SC [81, 88]. 

### 2.4. Lesion Tissue

FA within T2 lesions is usually decreased and MD increased [12, 18–21, 23, 89]. An overlap between MD and FA maps and T2 lesion distribution has been demonstrated in most MS phenotypes [27], with one exception being PPMS patients, in whom a discrepancy between regional WM diffusivity changes and T2-visible focal lesions has been found [69]. The absence of any overlap in PPMS between FA maps and T2 lesions [27] as well as of any correlation between diffusion metrics and lesion volume [90] lends support to the hypothesis that axonal damage and T2 lesions in this phenotype are, unlike those in other phenotypes, partially independent.

Pronounced FA and MD changes expressing both tissue damage and vasogenic edema have been detected in gadolinium-enhancing lesions (GEL) [24, 41, 89, 91, 92]. However, FA is more sensitive to pathological damage than MD [14, 62]. Indeed, in active lesions, FA values decrease according to the severity of tissue disruption [41], whilst MD values may increase, decrease, or be similar to those detected in chronic lesions [12, 19, 41, 89]. Acute demyelinating lesions exhibiting restricted diffusion, which have been described recently, are thought to be caused by the presence of inflammatory infiltrate or cytotoxic edema involving oligodendrocytes [93]. 

T1 hypointense lesions (black holes) [94], characterized by severe tissue injury, are associated with the most severe diffusion alterations because of their pathological characteristics [1, 12, 19, 41, 62, 89]. 

Since lesions have also been detected in GM, diffusion parameters have been investigated in this structure as well [95]. FA in GM lesions has, unlike that in WM, been found to increase. This finding may be due to the different histological characteristics of GM focal lesions, which present a higher level of activated microglia and greater loss of dendrites and axons along with a lower degree of inflammation than WM lesions [78, 96, 97]. 

## 3. DTI Metrics and Clinical Disability

Nonconventional techniques may detect microstructural changes and correlate with the clinical impairment more closely than usual MRI measures (i.e., T2 lesions, GEL, and T1 hypointensities), thus partially overcoming the so-called “clinical-radiological paradox” [98].

Diffusion abnormalities are more pronounced in patients with a long disease duration and severe neurological disability [54, 64, 71, 99], accurately reflecting the clinical condition. Attempts to correlate DTI metrics of the brain and SC with the most widely used clinical disability scale, that is, the Expanded Disability Status Scale (EDSS) [100], have yielded controversial results, with some studies finding a significant correlation [81, 101–104] not detected by others [6, 60]. The weak correlation between the EDSS and diffusion parameters may be due to several reasons: first of all, DTI measures, particularly when applied to NAWM, indicate pathologically affected, though still functioning, fiber tracts [6]; secondly, the EDSS is a global clinical index that is affected above all by the motor system and might consequently not be severely altered in the early disease stage; thirdly, when diffusion changes are evaluated in the whole brain, a clinical correlation with measures of disability is less likely to be found. Indeed, when diffusion was analyzed at the regional level, in specific tracts, more pronounced correlations with clinical features emerged; that is, the EDSS was correlated with DTI changes in motor tracts [105, 106] and with quantitative fiber tractography results [107].

Moreover, when correlations between regional DTI measures and clinical scales assessing specific clinical features were investigated, the role of microstructural damage in disability became more evident [108, 109]. In particular, oculomotor function impairment was associated with a focal DTI alteration in small brainstem fiber pathways [3]. In addition, diffusion abnormalities in the optic nerves were also significantly correlated with visual evoked potential parameters, suggesting that DTI metrics may be used as a surrogate measure of axonal damage [110, 111]. Diffusion measures in the optic nerve, optic radiation, and regional brain areas related to visual cortices were also associated with visual acuity [112, 113] and retinal nerve fiber layer thickness [113]. 

Regional diffusivity studies have also been used to find the anatomical substrates underlying the impairment of other functional systems. Tractography studies have demonstrated a correlation between MD and FA alterations in specific WM tracts, such as the corticospinal tract (CST) and the Corpus Callosum (CC), and motor disability [114–117]. Furthermore, cerebellar DTI abnormalities have been correlated with upper and lower limb disability [118]. Similarly, altered DTI parameters along the cerebellar connections and supratentorial associative WM bundles were correlated with balance impairment [119].

DTI can also reveal tract injury responsible for cognitive dysfunction in MS patients [71, 72, 108, 120, 121]. Focal abnormalities, particularly in the CC, have been related to calculation, sequence learning, and memory [121–126]. Moreover, several studies have detected correlations between cognitive impairment and diffusion metrics abnormalities in the posterior thalamic radiations [121] as well as fronto-subcortical fiber tracts [127] and the thalamus [77]. 

FA abnormalities in specific NAWM tracts have even been found to be significantly correlated with various cognitive abilities in pediatric patients in the early phase of disease [128]. 

Lastly, a strong correlation was found between DTI measures in the SC and disability, thus corroborating the role played by the pathological involvement of the spine in the clinical manifestations of the disease [86].

## 4. DTI Metrics and Nonconventional MRI

Diffusion studies have also been combined with other nonconventional MRI techniques, such as spectroscopic, magnetization transfer ratio (MTR), and functional MRI.

When MRI spectroscopy was applied together with DTI, the results were inconsistent. One early work did not find any correlation between DTI metrics and N-acetyl aspartate (NAA) values [129], thus suggesting that chronic metabolic dysfunction contributes to axonal pathology in MS. This result was not, however, confirmed in a more recent study, which reported correlations between diffusion metrics and NAA/Creatine ratios, thereby pointing to a link between microstructural and metabolic alterations [130]. This discrepancy is likely due to the fact that diffusion measures highlight changes induced by structural axonal loss, whereas NAA changes may be related to other, even transient, factors such as the functionality of neurons [129].

A multiparametric study, based also on MTR MRI, detected metabolic and diffusivity changes not related to MTR measures in the CC of CIS patients [131]. The widely reported lack of any correlation between diffusion metrics and MTR values [23, 44, 132, 133] suggests that these two methods may be sensitive to different pathological processes and may provide independent measures of damage [132].

Lastly, DTI has been used to quantify brain damage in functional MRI (fMRI) studies to investigate correlations between structural damage and functional changes. The results of most task-related fMRI studies support the idea that compensatory neuroplasticity is designed to maintain normal function in the presence of widespread microstructural damage. Indeed, altered DTI parameters have been found to correlate with an increase in fMRI activation during various tasks in MS patients [134–136]. In addition, the ultrastructural damage of specific WM tracts may affect cortical activity. For example, Lenzi et al. [136] found that MD values in the body of CC correlated with activation of the ipsilateral motor cortex during hand movements, thereby suggesting that functional changes in this area are related to the loss of transcallosal inhibitory fibres in MS [136]. More recent studies have correlated measures of anatomical and functional connectivity: in MS, distinct functional networks exhibit increases in functional connectivity despite widespread diffusivity abnormalities within WM [137].

DTI was used to investigate the relationship between WM damage and GM atrophy in CIS. Henry et al. (2009) measured diffusivity indexes in the thalamocortical tracts that connect WM lesions and the thalami. They found that both lesions and DTI values in thalamocortical tracts correlated with atrophy, which points to a direct relationship between WM lesions and thalamic atrophy [49]. By contrast, DTI metrics alterations in GM were not related to concomitant brain atrophy progression [138, 139].

## 5. Future Directions

Given their marked sensitivity in detecting structural tissue abnormalities in MS, DTI metrics have been used to monitor structural changes that occur during the course of this disease [73, 99, 140] in both WM and GM. The potential of DTI parameters as prognostic markers of disease evolution has also been evaluated. Unfortunately, the results are not conclusive, probably because of differences in the methods used to conduct these investigations. 

Although abnormalities in RRMS patients were found throughout the brain, no longitudinal diffusion changes were observed in the follow-up [141], nor were any longitudinal FA changes observed in CIS patients when they were studied again after conversion to MS [139]. As these findings suggest that WM damage occurs early but progresses slowly, the information yielded by a global diffusion assessment of normal appearing brain tissue may be of limited value as a means of following disease progression, at least at disease onset. By contrast, serial diffusion MRI images in PPMS patients have detected progressive NAWM changes, which proved to be related to both lesion volume and the development of clinical disability [142]. The role of DTI metrics as predictive markers has also been highlighted in other studies that have evaluated various MS phenotypes. Although agreement on this question is not unanimous [48, 138], some authors have demonstrated not only that altered regional NAWM metrics are predictive of clinical impairment [46, 117], of the development of new T2 lesions and atrophy [43, 131], and of the future risk of MS development [143] but also that more severe diffusivity brain abnormalities in WM and GM predict higher disability [78, 144]. DTI metrics also proved to be reliable as predictive markers of disease course when the SC was evaluated in patients with cervical relapses. Indeed, a lower RD in the lateral columns at baseline was associated with a better clinical outcome, with a greater decrease in RD being observed as patients improved clinically during follow-up [145]. 

Common guidelines are required for longitudinal DTI studies to overcome the existing discrepancies in such studies. Further regional trials designed to detect more focused MRI abnormalities, which may be used to monitor structural changes over time and, consequently, to shed light on clinical features, are warranted. Indeed, in order to be considered as an outcome measure in clinical trials, DTI parameters must be sensitive to change over time and must be highly reproducible and the sample size of the studied cohort must be appropriate to ensure the reliability of the results. The sample size needs to be planned on the basis of the statistical methods to be adopted, of the precision of the information required, and of the number of hypotheses to be tested, and in such a way as to take in account any missing values or drop-out patients. One interesting study conducted a power analysis to calculate reasonable sample sizes for longitudinal DTI studies. In brief, the authors of that study found that approximately 40 participants per arm were required for 1- to 2-year longitudinal DTI trials, though the number could vary according to the MS phenotypes and the anatomical region being evaluated [146].

## 6. Conclusions

Diffusion abnormalities in the NAWM and NAGM have been demonstrated in all phenotypes of MS, with microstructural changes being detected in early disease stages, even in early onset MS. Nevertheless, diffusion metrics appear to differ according to the MS phenotype and within different kinds of lesions. Since MS phenotypes display different diffusivity patterns, which may be due to specific pathological substrates, diffusion measures may represent useful markers of different MS subtypes. Moreover, when diffusion measures are combined with other MRI findings, they may provide complementary information on different types of pathological damage induced by MS. Lastly, given their high sensitivity in detecting structural tissue abnormalities, DTI measures have been proposed as prognostic markers of disease course and as a means of monitoring anatomical changes over time; further studies are warranted in this field to achieve more consistent results.

## Figures and Tables

**Table 1:** Schematic description of the main DTI parameters.

	Unit of measure	Formula	Object measured
FA	Scalar value ranging between 0-1	$\sqrt{\frac{1}{2}}\frac{\sqrt{{\left(\lambda_{1}-\lambda_{2}\right)}^{2}+{\left(\lambda_{2}-\lambda_{3}\right)}^{2}+{\left(\lambda_{3}-\lambda_{1}\right)}^{2}}}{\sqrt{\lambda_{1}^{2}+\lambda_{2}^{2}+\lambda_{3}^{2}}}$	Fibers directionality/axonal loss
MD	mm^2^/sec	(*λ*_1_ + *λ*_2_ + *λ*_3_)/3	Amount of water diffusion/myelin loss
AD	mm^2^/sec	*λ*_1_	Diffusivity parallel to the fibers/myelin and axonal content
RD	mm^2^/sec	(*λ*_2_ + *λ*_3_)/2	Diffusivity perpendicular to the fibers/myelin content

FA: fractional anisotropy; MD: mean diffusivity; AD: axial diffusivity; RD: radial diffusivity; mm: millimeters; sec: second.
